# Validation of an activity monitor for children who are partly or completely wheelchair-dependent

**DOI:** 10.1186/s12984-015-0004-x

**Published:** 2015-02-06

**Authors:** Carla FJ Nooijen, Janke F de Groot, Henk J Stam, Rita JG van den Berg-Emons, Hans BJ Bussmann, Fit for the Future Consortium

**Affiliations:** Department of Rehabilitation Medicine, Erasmus MC University Medical Center Rotterdam, P.O. Box 2040 , 3000 CA Rotterdam, The Netherlands; Research Group Lifestyle and Health, HU University of Applied Sciences, Utrecht, the Netherlands

**Keywords:** Activity monitor, Physical behavior, Physical activity, Wheelchair, Spina bifida, Cerebral palsy

## Abstract

**Background:**

Children who are wheelchair-dependent are at risk for developing unfavorable physical behavior; therefore, assessment, monitoring and efforts to improve physical behavior should start early in life. VitaMove is an accelerometer-based activity monitor and can be used to detect and distinguish different categories of physical behavior, including activities performed in a wheelchair and activities using the legs. The purpose of this study was to assess the validity of the VitaMove activity monitor to quantify physical behavior in children who are partly or completely wheelchair-dependent.

**Methods:**

Twelve children with spina bifida (SB) or cerebral palsy (CP) (mean age, 14 ± 4 years) performed a series of wheelchair activities (wheelchair protocol) and, if possible, activities using their legs (n = 5, leg protocol). Activities were performed at their own home or school. In children who were completely wheelchair-dependent, VitaMove monitoring consisted of one accelerometer-based recorder attached to the sternum and one to each wrist. For children who were partly ambulatory, an additional recorder was attached to each thigh. Using video-recordings as a reference, primary the total duration of active behavior, including wheeled activity and leg activity, and secondary agreement, sensitivity and specificity scores were determined.

**Results:**

Detection of active behaviour with the VitaMove activity monitor showed absolute percentage errors of 6% for the wheelchair protocol and 10% for the leg protocol. For the wheelchair protocol, the mean agreement was 84%, sensitivity was 80% and specificity was 85%. For the leg protocol, the mean agreement was 83%, sensitivity was 78% and specificity was 90%. Validity scores were lower in severely affected children with CP.

**Conclusions:**

The VitaMove activity monitor is a valid device to quantify physical behavior in children who are partly or completely wheelchair-dependent, except for severely affected children and for bicycling.

## Background

Persons who are wheelchair-dependent are known to be less physically active compared to able-bodied persons [1]. Moreover, compared to persons with other chronic diseases, persons who are wheelchair-dependent have less active lifestyles [1]. This unfavorable physical behavior results in low physical fitness levels and increased risk for secondary conditions, including cardiovascular diseases, difficulties with participation and lower quality of life [2-5]. Independent of physical activity, a person with a large amount of sedentary time [6] may still be at risk for poor health outcomes. Consequently, besides meeting physical activity guidelines it is also recommended to limit the amount of sedentary time [7].

Little is known about physical behavior of children who are partly or completely wheelchair-dependent. Two studies with limited sample size showed that in adolescents and young adults with spina bifida (SB) or cerebral palsy (CP) who are wheelchair-dependent, objectively measured physical activity level was 56% [8] and 31% [9] lower than in able-bodied controls. A larger study confirmed these findings, reporting that adolescents and young adults with SB who were wheelchair-dependent were more than three times less physically active compared to able-bodied controls [10].

Physical behavior, like other behaviors, is often learned during childhood, with increasing evidence suggesting that healthy active children become active healthy adults [11-13]. Therefore, the recent literature suggests that interventions should ideally start before the transition from youth to adulthood [14]. Therefore, monitoring and optimizing physical behavior are increasingly important in the field of pediatric rehabilitation. This new approach requires valid and reproducible outcomes to assess and monitor physical behavior and its changes in children who are wheelchair dependent. Self-reported physical behavior is known to have limited validity for quantifying actual physical behavior [15-17]. Objective physical behavior data can be obtained using accelerometer-based activity monitors.

For ambulatory persons, a wide range of activity monitors are available [18]. For persons who are wheelchair dependent, the choice of devices is limited and restricted to two types: wheelchair-mounted accelerometers [19,20] and body-fixed accelerometers [21-23]. Wheelchair-mounted accelerometers have some important limitations. For example, a wheelchair-mounted accelerometer will register physical activity when a wheelchair is pushed by another person, and physical activity performed when changing to another aid (e.g., handcycle) will not be registered. Furthermore, in persons who are partly wheelchair-dependent, activities using the legs will not be registered. An activity monitor with body-fixed accelerometers does not have these limitations. Multiple body-fixed accelerometers may have the disadvantage from the viewpoint of applicability, but will enable to detect and distinguish both leg and wheelchair activities in persons who are partly or completely wheelchair-dependent.

One available activity monitor with multiple body-fixed accelerometers is the VitaMove activity monitor. Accelerometer signals from the trunk and wrists allow the automatic detection of physical behavior categories such as wheelchair propulsion, handcycling, sitting and lying down. If leg sensors are added for a person who is partly wheelchair-dependent, activities involving the legs (e.g., standing, walking and cycling) can be detected and distinguished as well. The VitaMove activity monitor enables the collection of detailed information on physical behavior, including the amount, intensity and pattern of different types of physical activity and sedentary time.

In adults, both ambulatory and wheelchair-dependent, the predecessor of this activity monitor has demonstrated validity for quantifying physical behavior [22,24,25]. However, validity in persons who are partly wheelchair-dependent and validity in children has not been described previously. Because children show different movement patterns [26] characterized by both shorter movement bouts and different types of activities (e.g., crawling), it is necessary to validate the VitaMove activity monitor with a protocol adapted for children. Therefore, the purpose of this study was to assess the validity of the VitaMove activity monitor to quantify physical behavior in children who are partly or completely wheelchair-dependent. We focused on children with SB and CP because these are the two largest groups in pediatric rehabilitation [27].

## Methods

### Participants

Children with SB and CP were recruited and selected based on availability at the HU University of Applied Sciences and at Rehabilitation Center De Hoogstraat, both located in Utrecht, The Netherlands. Inclusion criteria were: 1) age 6 to 18 years; 2) partial or complete wheelchair-dependence, 3) experience with wheelchair use and 4) ability to propel oneself using the handrims of a wheelchair. The study was approved by the Medical Ethics Committee of Erasmus MC, University Medical Center Rotterdam. Informed consent was obtained from all participants. Age, diagnosis, Hoffer classification (for persons with SB) [28] and Gross Motor Functioning Classification System (GMFCS) level (for persons with CP) [29] were recorded.

### Testing procedure

On one occasion, participants performed a series of consecutive activities according to a standard protocol in a natural setting, either at their home or at their school. The protocol consisted of several activities thought by experienced physical therapists to be representative for everyday life in children who are partly or completely wheelchair-dependent. All children completed a wheelchair protocol consisting of several activities while sitting in a wheelchair. If possible, children also completed a protocol with activities using their legs (leg protocol) (Table 1). Participants performed the activities using their own mobility aids and were instructed to perform the activities at their own pace and in their own manner. They performed only those activities that they were able to perform and that they performed regularly. Total measurement time was about 25 minutes per participant. Simultaneous measurements were made using the VitaMove activity monitor and video recording (as the reference method) during protocol performance.

**Table 1:** **Wheelchair protocol and leg protocol**

**Wheelchair protocol**	**n**	**Total duration (s)**	**Motility (g)** ^**1**^
Donning a coat	12	618	41.33
Handcycle, slow^2^	7	567	43.43
Handcycle, comfortable^2^	6	390	76.50
Handcycle, fast^2^	6	318	161.50
Wheelchair, slow^2^	11	718	82.18
Wheelchair, comfortable^2^	12	812	104.17
Wheelchair, fast^2^	11	601	196.28
Play basketball	11	1389	76.10
Being pushed without arm movement	12	751	23.67
Being pushed with random arm movement	12	714	47.67
Open door, drive through door and close door	10	218	44.78
Make a drawing	12	1004	8.25
Play a game on a mobile phone	12	1102	6.25
Manoeuvring through the kitchen	11	373	52.81
Leaf through a magazine	12	827	12.42
Lying down	12	832	10.92
In between activities	12	2756	29.42
**Leg protocol**	**n**	**Total duration (s)**	**Motility (g)** ^**3**^
Standing	4	293	6.25
Walking (on comfortable speed)	5	301	62.40
Crawling	2	233	57.50
Bicycling	2	250	45.00
In between activities	5	340	32.80

^1^Motility for all participants determined for 3 sensor configuration.

^2^Slow, comfortable and fast were all self-chosen speeds.

^3^Motility determined for 5 sensor configuration.

### VitaMove activity monitor

**Figure 1:**
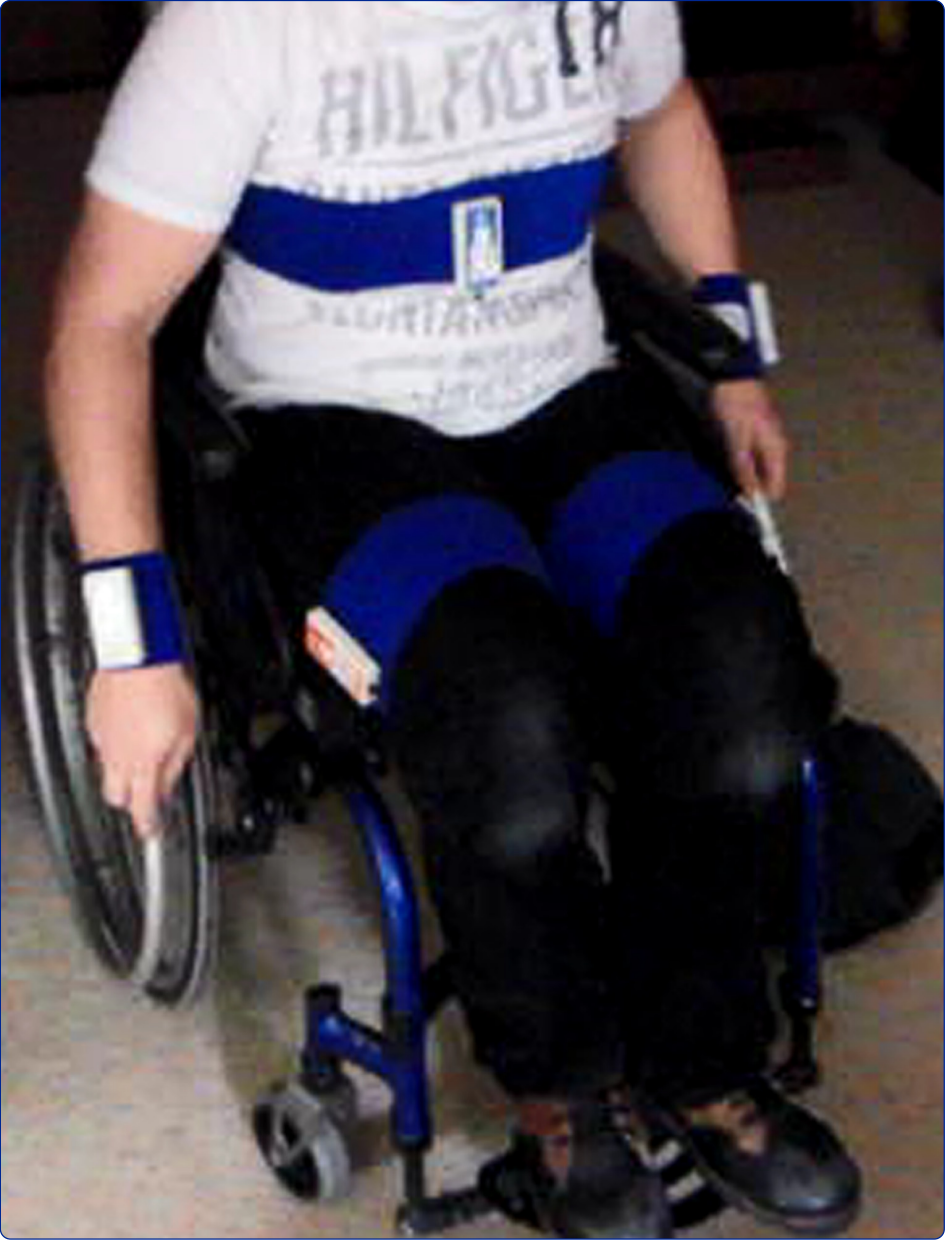
**The VitaMove activity monitor.**

The VitaMove activity monitor is a commercially available ambulatory monitoring system (2 M Engineering, Veldhoven, The Netherlands) with body-fixed, three-axis accelerometers (Freescale MMA7260Q, Denver, USA). The VitaMove activity monitor is the successor of the Vitaport/Rotterdam Activity Monitor, which is previously validated and applied, also in wheelchair users [22,25]. The VitaMove activity monitor can be worn for 7 days, and since it is not waterproof has to be removed during swimming or bathing. The wheelchair detection version of the predecessor model consisted of five accelerometers connected to a data recorder carried in a belt around the waist. The new version, used in the current study, is wireless (Figure 1), and the analysis software has been translated to a new platform. The system consists of three or five recorders that are wirelessly connected and synchronized every 10 seconds. In children who were completely wheelchair-dependent, one recorder was attached to the sternum and one to the anterior side of each wrist using specially developed belts. In children who were partly ambulatory, an additional recorder was attached to each mid-thigh on the lateral side. Accelerometer signals from each recorder were sampled with a frequency of 128 Hz and stored digitally on a micro Secure Digital memory card. Measurements were uploaded to a computer for kinematic analysis using VitaScore Software (VitaScore BV, Gemert, the Netherlands). VitaMove data analysis, which is an automated process, consisted of three parts: (1) feature processing, (2) activity detection and (3) post-processing.

During feature processing, feature signals were derived from each measured signal. First, angular signals were created by low-pass filtering the measured signals and converting them to an estimate of the angular position of the segment to which the sensor was attached (expressed in degrees). Second, a motility signal was created by high-pass filtering, rectifying and smoothing the data. The motility signal depends on the variability of the measured signal around the mean and is expressed in the acceleration unit (g). The third signal was the frequency signal, based on Fast Fourier Transform applied on a band-pass-filtered derivative of the measured signals. Additionally, phase feature signals were created by determining the phase between the two legs and between the two arms. The time resolution was one second for all feature signals.

**Figure 2:**
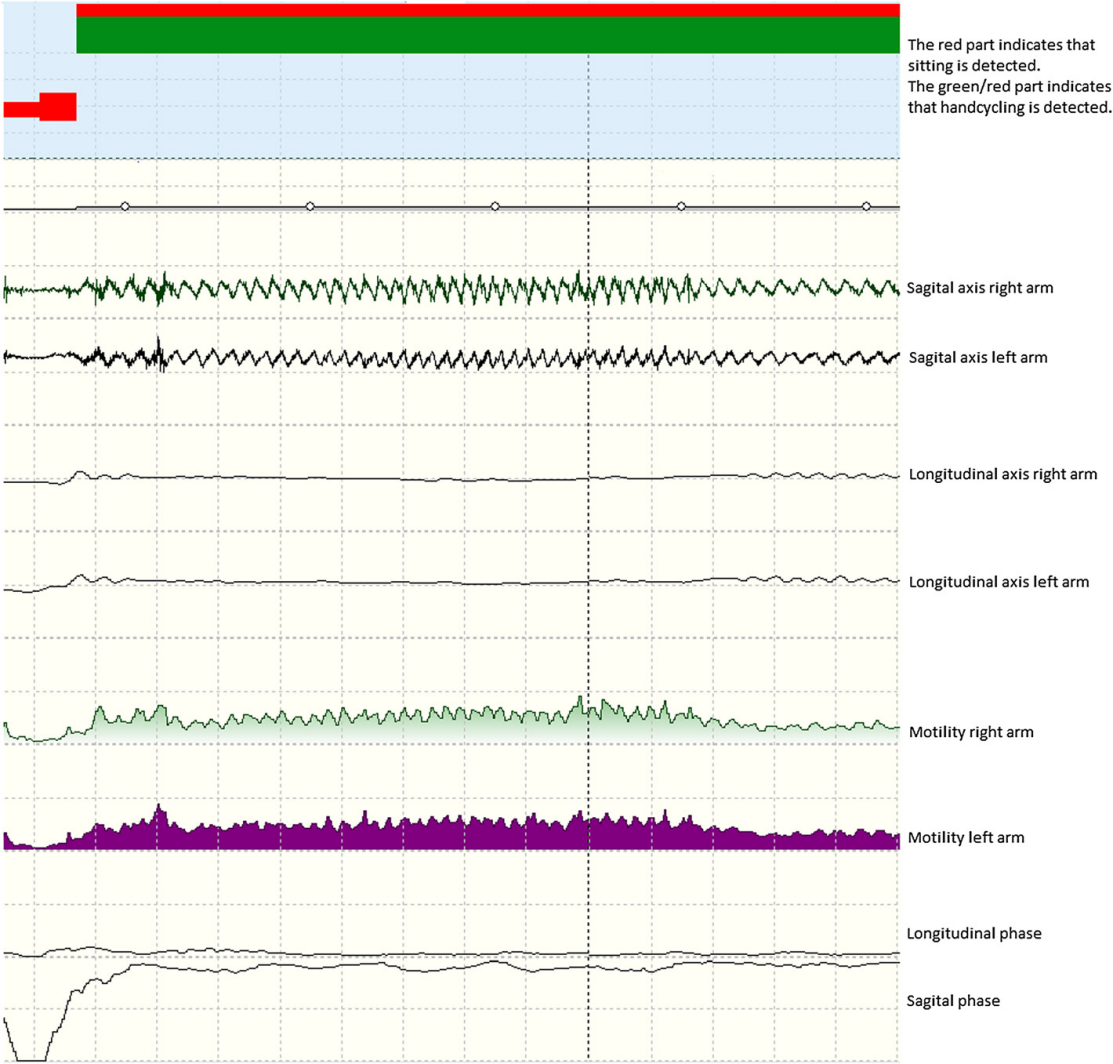
**Feature signals during sitting and handcycling.**

Based on these features and a minimal distance routine, a body posture or movement was selected from a large set (>40) of body (sub)postures and (sub)movements every second. Then, subcategories were merged into main categories (e.g., lying, sitting, standing, walking, running, bicycling, crawling, general movement, wheelchair propulsion or handcycling). During post-processing, short-lasting activities (<5 seconds) were disregarded. The same routines and rules were applied in all participants. Furthermore, body motility (in g*100), a measure of movement intensity [30], was determined. In persons who were completely wheelchair-dependent, body motility was the sum of trunk motility and the mean of both arm motility signals. In persons who were partly wheelchair-dependent, the mean motility signal of the legs was averaged with the mean motility signal of the arms and added to the sum of the trunk motility. For descriptive reasons, mean body motility was determined for all protocol activities (Table 1). Figure 2 shows an example of feature signals for a short period of sitting followed by handcycling.

### Reference method

Video recordings were made using a handheld camera. All video recordings were analysed independently from the VitaMove activity monitor output by two persons, using a time resolution of one second. Samples on which the two persons disagreed were reviewed together to determine a final score. The continuous VitaMove activity monitor output was compared to the synchronized, continuous video-analysis output, both using a time resolution of one second.

**Figure 3:**
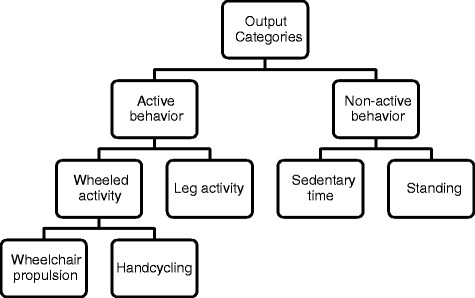
**Flow chart of output categories.**

For both the VitaMove activity monitor and the video scores, every second was assigned to one of the categories as shown in Figure 3. Wheelchair propulsion was defined as independent propulsion of oneself to another location, while sitting in a wheelchair, as a result of arm power. This included maneuvering. Handcycling was defined comparable to wheelchair propulsion but while sitting in a wheelchair with an add-on hand-cycle or while sitting or lying in a hand-cycle. Leg activity included walking, bicycling and crawling. Sedentary time included sitting and lying down.

### Data analysis

We analysed our data using an approach characterized by increasing detail. We started with our primary outcome: the difference, in seconds and as a percentage, in the total amount of registered active behavior between the VitaMove activity monitor and video. For this analysis we used the output categories: active behavior and non-active behavior. We determined the difference per participant and calculated the sum of errors and mean absolute percentage error separately for the wheelchair and the leg protocol. We considered that a difference less than 10% indicated good validity.

Secondary, agreement, sensitivity and specificity were assessed, for the wheelchair protocol and the leg protocol separately. For this analysis we distinguished between the following output categories: wheeled activity, leg activity, sedentary time and standing.

Agreement: the percentage of agreement between all samples of video and VitaMove activity monitor. Agreement was calculated according to: ((Number of identical seconds of video and VitaMove)/(total number of seconds))*100.

Sensitivity: the degree in which the video categories wheeled activity or leg activity were detected correctly by the VitaMove activity monitor. Sensitivity was calculated according to: ((Number of identical seconds of video and VitaMove for video category “wheeled activity or leg activity”)/(total number of seconds for this video category))*100

Specificity: the degree in which the activity monitor was able to exclude the video categories wheeled activity and leg activity correctly. Specificity was calculated according to: ((Number of seconds in which “wheeled activity or leg activity” were not detected by the VitaMove for video category other than “wheeled activity or leg activity”)/(total number of seconds for video category other than this video category))*100

An outcome of >90% was considered excellent, 80% to 90% good, 70% to 80% moderate and less than 70% weak.

As a supplement, error detections of the VitaMove activity monitor were analysed to the most detail in output categories (wheelchair propulsion, handcycling, leg activity, sedentary time and standing). Finally, we assessed during which protocol activity the VitaMove activity monitor had the most detection errors. We determined the contribution of errors within each protocol activity and compared these to the total amount of errors within the wheelchair protocol and leg protocol, expressed as a percentage, and corrected for the duration of each protocol activity.

## Results

### Participants

Twelve children participated, including nine with SB and three with CP. Participant characteristics are shown in Table 2. The mean age was 14 ± 4 years. Five participants were measured with five recorders and performed both the wheelchair and leg protocol. The other seven participants were measured with three recorders and performed only the wheelchair protocol.

**Table 2:** **Participants’ characteristics**

**Participant**	**Gender**	**Age**	**SB/CP**	**Hoffer/GMFCS**	**Number of sensors**	**Handcycle**	**Stand**	**Walk**	**Crawl**
1	M	12	SB	Community	5	Bike	√	√	x
2	F	13	SB	House-hold	5	√	√	√	√
3	F	15	SB	Community	5	Bike	√	√	x
4	F	16	SB	House-hold	5	√	√	√ + chair	x
5	M	17	SB	House-hold	5	x	x	stairs + chair^1^	√
6	M	10	SB	Non-functional	3	√	x	x	x
7	M	17	SB	Non-functional	3	√	x	x	x
8	M	18	SB	Non-functional	3	√	x	x	x
9	F	18	SB	Non-functional	3	√	x	x	x
10	F	6	CP	IV	3	x	x	x	x
11	F	13	CP	IV	3	x	x	x	x
12	M	17	CP	III	3	√	x	x	x

M = male, F = female, SB = Spina Bifida, CP = Cerebral Palsy, L = lumbar, Th = thoracic.

GMFCS = Gross Motor Function Classification System.

^1^Only able to walk on stairs and while sitting on a chair.

For the wheelchair protocol (n = 12), the mean duration was 19.4 ± 3.5 minutes and the mean motility was 59.86 ± 53.27 g. For the leg protocol (n = 5), the mean duration was 4.7 ± 1.2 minutes and the mean motility was 40.79 ± 22.48 g.

Table 3 shows the results on our primary outcome: the difference in total amount of registered active behavior between the VitaMove activity monitor and video. For the wheelchair protocol, the absolute percentage error was 6% (range −9 to +20%), which indicates excellent validity (<10%). One participant’s activity was overestimated by more than 10%. For the leg protocol (n = 5), the validity was found to be good, with an absolute percentage error of 10% (range −25 to +7%). Two participants’ activity was underestimated by more than 10%.

**Table 3:** **Difference in registered active behaviour between VitaMove activity monitor and video**

**Participant**	**Wheelchair protocol**			**Leg protocol**		
	**Total duration**	**Activity difference**		**Total duration**	**Activity difference**	
	**seconds**	**seconds**	**%**	**seconds**	**seconds**	**%**
1	830	+162	+20	223	−37	−17
2	1122	+80	+7	271	0	0
3	826	−78	−9	388	−97	−25
4	1382	7	0	214	+14	+7
5	1003	+91	+9	321	−5	−2
6	1396	+4	0			
7	1392	+53	+4			
8	1259	+43	+3			
9	1390	−61	−4			
10	1079	−39	−4			
11	1118	−85	−8			
12	1202	−78	−7			
*Sum*	*13999*	*+99*	*+11*	*1417*	*−125*	*−37*
*Absolute % error*			*6*			*10*

+ indicates overestimation and – underestimation of active behaviour by the VitaMove activity monitor.

Table 4 shows the agreement, sensitivity and specificity for the wheelchair and leg protocols. For the wheelchair protocol, overall agreement, sensitivity and specificity were excellent. The agreement score was excellent (>90%) for one participant, good for nine participants and moderate for two participants. Three participants had sensitivity scores and one participant had a specificity score that was considered weak (<70%). For the leg protocol, overall agreement and specificity were good and sensitivity was moderate. Agreement was weak for one participant and sensitivity was weak for two participants completing the leg protocol.

**Table 4:** **Agreement, sensitivity, and specificity for wheeled activity and leg activity**

	**Wheelchair protocol**			**Leg protocol**		
**Participant**	***Agreement***	***Sensitivity***	***Specificity***	***Agreement***	***Sensitivity***	***Specificity***
1	80	100	71	76	67	91
2	81	86	78	96	96	97
3	77	99	66	63	52	83
4	87	83	90	93	100	91
5	85	90	83	85	78	90
6	82	72	87			
7	81	79	82			
8	86	86	86			
9	79	60	88			
10	89	65	96			
11	91	67	99			
12	87	77	95			
*Mean*	*84*	*80*	*85*	*83*	*78*	*90*

When analysing error detections to the most detail in output categories it was found that in the wheelchair protocol errors occurred mostly for the categories wheelchair propulsion and handcycling, which were both determined as sedentary time, and the other way around. A total of 1299 (1000 plus 299) seconds of sedentary time was detected as wheelchair propulsion or handcycling respectively and 922 (548 plus 374) seconds of wheelchair propulsion and handcycling were detected as sedentary time by the VitaMove activity monitor. This difference of 377 seconds corresponds to 29% of these detection errors of sedentary time and 41% of these detection errors of wheelchair propulsion.

Analyses of the data per protocol activity showed that, in the wheelchair protocol, most detection errors occurred during the protocol activity handcycling. When taken together the errors in the protocol activities of slow, comfortable and fast handcycling, this accounted for 36% of all detection errors. Other activities with relatively high detection error rates were: open door/close door (13%), play basketball (11%), being pushed with random arm movement (10%) and getting something from the kitchen and returning (9%). Other protocol activities each accounted for less than 5% of errors. For the leg protocol, most errors occurred during the protocol activity bicycling (64%). Crawling accounted for 10% of errors, walking for 9% and standing for 3%. The remaining 14% of errors occurred between protocol activities.

## Discussion

This is the first study to assess the validity of an activity monitor in a non-laboratory setting for children who are partly or completely wheelchair-dependent. For children who are wheelchair-dependent the VitaMove activity monitor consists of one recorder on the sternum and two on the wrists and in children who are partly ambulatory, an additional recorder can be attached to each thigh. The results indicate that the VitaMove activity monitor is a valid device to quantify physical behavior in children who are partly or completely wheelchair-dependent. Primary, this activity monitor succeeded in accurately measuring the total duration of active behavior. Secondary, overall agreement, sensitivity and specificity scores were good.

Previous studies have also reported good validity for the use of an activity monitor in adults who are wheelchair-dependent [19-22]. A previous study determined sensitivity, specificity and agreement as outcome measures [22]. That study concerned the predecessor of the VitaMove activity monitor and showed somewhat higher agreement, sensitivity and specificity scores (92%, 87% and 92%, respectively). The absolute difference in duration of wheelchair propulsion between the activity monitor and the video analysis was somewhat lower in the previous study (1073 seconds on a total of 16039 seconds: 7%). Different in the present study is that we included maneuvering as part of wheelchair propulsion, whereas the previous study excluded samples classified as maneuvering because they considered it to be a mixture of wheelchair propulsion and other activities. Protocol activities of playing basketball, opening and closing the door and maneuvering through the kitchen were associated with a relatively large amount of detection errors during the current study, partly because of the relatively large amount of maneuvering involved in these activities. During maneuvering, the most common error was that the VitaMove activity monitor detected sitting instead of wheelchair propulsion. Outcome measurements in other previous studies were different and therefore less comparable. Two studies on a different wheelchair-mounted activity monitor found a point-by-point accuracy of over 90% [19] and an intra-class correlation coefficient of 0.98 for duration of movement [20]. Another study reported a classification accuracy of 96% for the activities resting, propulsion, arm ergometry and deskwork [21].

The VitaMove activity monitor did not show considerable overestimation or underestimation of active behavior. The distinction between active and non-active behaviour is most important with regard to health benefits [3]. A more detailed analysis of the errors implied that a substantial portion of detection errors might be caused by timing problems, meaning that the activity category was correctly detected by the VitaMove activity monitor but not at the exact same second as the activity occurred in the video. This discrepancy is possibly caused in part by post-processing, in which activities less than five seconds were disregarded. For registering physical activity and sedentary time during multiple day measurements it is an important finding that the amount of wheeled activity falsely categorized as sedentary time and the amount of sedentary time falsely categorized as wheeled activity were similar. Therefore, the consequences of error detection in registering total active behaviour during multiple day measurements seem to be minimal.

Handcycling and bicycling showed relatively the largest amount of detection errors. This can be partly explained by when children were not propelling the pedals but did sit on their handcycle or bicycle and were still moving due to earlier propulsion, the video was scored as (hand)cycling. However, this was not correctly detected by the VitaMove activity monitor since children were not moving their legs or arms at that moment. Furthermore, the software program includes assumptions that handcycling and bicycling are characterized by arm or leg movement that is nearly or completely in phase or out of phase. For this group of children with partial or complete wheelchair-dependence and severe movement disorders, these activities were not always this fluent. The VitaScore software does have an option providing manual override of the automatic detection. Although the recommendation should be to manually override as little as possible, it should be an option. Manual correction during post-processing is only in rare cases an option to consider, this will be in most cases measurements of the most severely affected children. This option seems feasible for handcycling and bicycling because these activities are relatively easy to visually distinguish based on feature signals, especially because they concern longer duration activities, see Figure 2 for an example of the feature signals of sitting and handcycling. For using the VitaMove activity monitor to perform physical behaviour measurements of multiple days, we made strict guidelines with recommendations for manual correction of longer periods of handcycling and bicycling that are not automatically detected. Guidelines include: the phase signal is the only non-corresponding feature and that the period of handcycling or bicycling is at least one minute. Using these guidelines should result in fewer detection errors for handcycling and bicycling. In the current study, these guidelines would have increased the sensitivity for the wheelchair protocol in participant 6 from 72% to 92% and in participant 9 from 60% to 88%. The guidelines also would have increased sensitivity for the leg protocol in participant 1 from 67% to 100% and in participant 3 from 52% to 98%.

When performing the protocol activity “being pushed with random arm movement,” participants were asked to make arm movements without any further instructions. There were a relatively large number of error detections during this protocol activity, partly because two children made arm movements that mimicked handcycling or wheelchair propulsion. This behavior by participants 1 and 3 partly explains the relatively low specificity for these children in the wheelchair protocol. When applying the VitaMove activity monitor for multi-day measurements, we assume that performance of this type of arm movement will be uncommon.

Furthermore, participants 10 and 11 had relatively low sensitivity for wheeled activities (65% and 67%, respectively). These participants were both diagnosed with CP classified as GMFCS level IV. Compared to less severely affected participants, these two children moved more slowly and their movements were smaller [31]. Therefore, physical behavior measurement results from the VitaMove activity monitor in children with GMFCS level IV should be interpreted with caution. Because our sample included only three participants with CP and only two participants with CP with GMFCS level IV, further study in this subgroup is necessary.

### Limitations

Although a strength of this study was that it was performed in a non-laboratory setting, we had to select protocol activities. Although protocol activities may be representative for type of physical behavior, they are not representative of duration of these behaviors in daily life. Furthermore, our sample size was limited and our sample was heterogeneous in age and in severity of the disorder. Furthermore, not all children were able to perform all protocol activities. For crawling and bicycling, the validation was limited to two persons; therefore, these activities require further study.

## Conclusion

We conclude that the VitaMove activity monitor is a valid device to quantify total physical behavior in children who are partly or completely wheelchair-dependent. Despite errors in detection of detailed categories, active and non-active behaviour were distinguished accurately, which is most important with regard to health benefits. Further study is necessary to determine whether the use of this activity monitor is appropriate for severely affected children with CP. Future studies should validate these findings and assess clinical feasibility in real life assessment of physical behavior in children who are wheelchair-dependent.

## Abbreviations

CP: Cerebral palsy
GMFCS: Gross motor functioning classification system
SB: Spina Bifida
